# Prognostic value of circulating tumor cells detected with the CellSearch system in esophageal cancer patients: a systematic review and meta-analysis

**DOI:** 10.1186/s12885-020-07059-x

**Published:** 2020-06-22

**Authors:** Yiding Li, Guiling Wu, Wanli Yang, Xiaoqian Wang, Lili Duan, Liaoran Niu, Yujie Zhang, Jinqiang Liu, Liu Hong, Daiming Fan

**Affiliations:** 1 State key Laboratory of Cancer Biology and National Clinical Research Center for Digestive Diseases, Xijing Hospital of Digestive Diseases, Fourth Military Medical University, 127 Changle West Road, Xi’an, Shaanxi Province 710032 PR China; 2 School of Aerospace Medicine, Fourth Military Medical University, Xi’an, 710032 China

**Keywords:** Circulating tumor cells, Esophageal carcinoma, Chemotherapy, Prognosis, Meta-analysis

## Abstract

**Background:**

Esophageal carcinoma (EC) is the seventh-most prevalent tumor in the world, which is still one of the primary causes of tumor-related death. Identifying noteworthy biomarkers for EC is particularly significant in guiding effective treatment. Recently, circulating tumor cells (CTCs) in peripheral blood (PB) were intensively discussed as prognostic markers in patients with EC. However, an ongoing controversy still exists regarding the prognostic significance of CTCs determined by the CellSearch system in EC sufferers. This meta-analysis was designed to approach this topic.

**Methods:**

We systematically conducted searches using PubMed, Medline, Web of Science and the Cochrane Library for relevant studies, which were published through February 20, 2020. Using the random-effects model, our study was performed in Review Manager software, with odds ratios (ORs), risk ratios (RRs), hazard ratios (HRs) and 95% confidence intervals (CIs) as the effect values.

**Results:**

Totally 7 articles were finally included in this study. For clinicopathological characteristics, the pooled results on TNM stage indicated that the III/IV group had higher rate of CTCs compared with the I/II group (OR = 1.36, 95% CI: 0.68–2.71, I^2^ = 0%). Incidence of CTCs was higher in patients with T3/T4 stage (OR = 2.92, 95% CI: 1.31–6.51, I^2^ = 0%) and distant metastasis group (OR = 5.18, 95% CI: 2.38–11.25, I^2^ = 0%) compared to patients with T1/T2 stage or non-metastatic group. The pooled analysis revealed that CTC positivity detected in EC patients was correlated with poor overall survival (OS) (HR = 2.83, 95% CI:1.99–4.03, I^2^ = 0%) and relapse-free survival (RFS) (HR = 4.71, 95% CI:2.73–8.13, I^2^ = 0%). When pooling the estimated RR, a poor therapeutic response to chemoradiotherapy was discovered in patients with CTC positivity (RR = 1.99, 95% CI:1.73–2.29, I^2^ = 60%).

**Conclusions:**

In summary, our meta-analysis demonstrated that CTCs positivity determined by the CellSearch system are correlated with the prognosis of EC patients and might indicate a poor therapeutic response to chemotherapy in EC patients.

## Background

Esophageal carcinoma (EC), one of the most frequent malignant tumor, was the seventh-most prevalent tumor (572,000 new cases) and the sixth primary cause of tumor death (509,000 deaths) in the world [1, 2] with a 5-year survival rate of 18 to 25% after diagnosis [3]. The most frequent subtypes of EC are squamous cell carcinoma (SCC) and adenocarcinoma (AC) that have a high incidences in Asian countries and in Western countries, respectively [4]. Despite advances in diagnostic and therapeutic modalities against EC, locoregional recurrence and distant metastasis remain significant problems. Due to the difficulty of identifying the patients with occult metastasis, even if metastasis were not detected in patients after undergoing surgery, they may still die of tumor recurrence at an early stage [5, 6]. Currently, EC spreading or metastasis could not be detectable early by radiological and endoscopic imaging techniques. Thus, early diagnostic markers for EC are urgently needed.

An important step in tumor metastasis is that tumor cells are shed from the primary tumor to the vasculature, where they can spread to other organs. Thus, for a deeper understanding of tumor metastasis and for the earlier detection of tumors, circulating tumor cells (CTCs) which are tumor cells detached from a primary tumor and then entered into the blood circulation, have been regarded as prognostic markers. Their relevance has been investigated in several scientific research studies [7], the first of which was described in 1869 by Prof. Ashworth [8]. The results of these studies show that CTCs, as a new and effective diagnostic and prognostic biomarker, have gradually been accepted to monitor tumor recurrence and treatment effect, to determine therapeutic strategies, and to predict the survival of patients [9] because of their advantages as an earlier, more reproducible, more reliable, and accurate prognostic indicator for disease status compared with current imaging methods [10].

A considerable amount of studies have demonstrated that the CTCs presenting in the peripheral blood (PB) indicate a poor prognosis in patients with EC [11, 12]. The withdrawal of PB is more convenient and less risky for patients, with comparable repeatability. With the further development of the CellSearch system, the ability to detect CTCs has become more reliable for certain metastatic tumors [13]. The CellSearch® system (Menarini Silicon Biosystems, Castel Maggiore, BO, Italy), a CTCs detection method based on immunological assay with the epithelial cell adhesion molecule (EpCAM), was placed on the market by Veridex Corporation (Warren, NJ) in 2004, which is currently the first and only FDA-approved CTCs assay for monitoring colorectal, breast, prostate tumors, etc. [14]. And the prognostic significance of CTCs determined using the CellSearch system has been summarized by previous studies in sufferers with colorectal, gastric and breast tumors [15–18]. However, an ongoing controversy exists regarding the significance of the CellSearch system-detected CTCs in predicting the prognosis of EC patients. Thus, it requires to provide more accurately prognostic relevance based on the available data of CTCs determined by CellSearch system in EC patients.

Considering the current controversies regarding the significance of the CellSearch system-detected CTCs in prognosis of EC patients, in our study, we systematically analysed data obtained in published literatures and summed up the potential clinicopathological and prognostic significance of the CellSearch system-detected CTCs in EC patients.

## Methods

### Search strategy

We systematically searched PubMed, Medline, Web of Science and the Cochrane Library for relevant studies, which published through February 20, 2020. The following key words were used: “Circulating tumor cells”, “CTCs”, “CellSearch system” and “esophageal cancer”. We used the following strategy: ((((((((Esophagus tumor) OR Esophageal tumor) OR Esophageal Cancer) OR Esophagus Cancer) OR Esophagus Neoplasm) OR Esophageal Neoplasms) OR “Esophageal Neoplasms”[Mesh]) AND ((((((((“Neoplastic Cells, Circulating”[Mesh]) OR occult tumor cells) OR isolated tumor cells) OR disseminated tumor cells) OR circulating neoplastic cells) OR circulating tumor cells) OR CTC) OR circulating tumor cells detection)) AND CellSearch system.

### Eligibility criteria and quality assessment

To be included in the meta-analysis, articles were selected based on the following criteria: (i) the articles only using the CellSearch system to detect CTCs and investigate the prognostic significance of CTC in EC patients; (ii) the article reported at least one noteworthy outcome indicator of CTCs, or the outcome could be calculated, based on data extracted from the published data; and (iii) the samples were collected from peripheral blood. Articles were excluded based on the following criteria: (i) the article was published in languages other than English; (ii) the number of EC patients and samples was less than 10; (iii) samples were collected from lymph nodes, bone marrow, or the abdominal cavity; (iv) non-human experiments; (v) reviews, case reports, comments, letters, and meeting records; (vi) EC and CTCs were not studied; and (vii) unable to obtain enough data through article reports and data calculations.

We evaluated the quality of the included literature with the Newcastle-Ottawa Scale (NOS), recommended by the Cochrane Library [19], according to three categories: (i) study group selection; (ii) comparability of groups; and (iii) outcome of interest. The full score was 9, and 1–4 points indicated low-quality, while 5–9 points were considered high-quality.

### Data extraction

Two reviewers independently used a standardized form to extract the data from the included studies: first author’s name, publication year, country of patients, characteristics of patients (number, sex, age), sampling time, detection markers, detection rate, histology, prognostic value, hazard ratio (HR) and disease control rate (DCR) of chemotherapy, and any disputes or differences were settled by a third independent investigator. For studies with multiple arms, each arm was considered an independent data set. The tumors DCR were evaluated in accordance with the Response Evaluation Criteria in Solid Tumors (RECIST) guideline [20]. The DCR is calculated as (complete response [CR] + partial response [PR] + stable disease [SD])/ (complete response [CR] + partial response [PR] + stable disease [SD] + progressive disease [PD]).

### Statistical analysis

We used Review Manager software (RevMan, version 5.3, The Nordic Cochrane Centre, The Cochrane Collaboration, London, UK) to analyze the data in our meta-analysis. The estimated odds ratios (ORs) from the included studies were used to assess the association between CTC detection and different clinicopathologic features of EC. To statistically assess the prognostic effects of CTCs, we extracted the HR and 95% confidence interval (CI) of overall survival (OS) and relapse-free survival (RFS) from the included studies. If HRs, 95% CIs, or P-values were not directly provided in the original literature, the estimated HR was used to assess prognostic effects based on the method described by Tierney et al. [21], and HR > 1 reflects further disease progression or more deaths in the patients with CTC positivity. Furthermore, the estimated risk ratio (RR) was calculated to assess the DCR. We pooled the extracted HRs together in Review Manager. All statistical values were combined with 95% CIs, and all P values were two sided whose threshold was considered statistically significant when it was less than 0.05. Heterogeneity among the studies was tested using Cochran Q test and I^2^ statistic. Significant heterogeneity was considered when P ≤ 0.1 or I^2^ ≥ 50% [22], and in these cases, a random-effects model was used. Simultaneously, according to the differences in the data retrieved, subgroup analyses were performed, such as for the age of patients, sex of patients, histology, and clinicopathological significance. Publication bias was evaluated using a funnel plot.

## Results

### Study characteristics

**Fig. 1:**
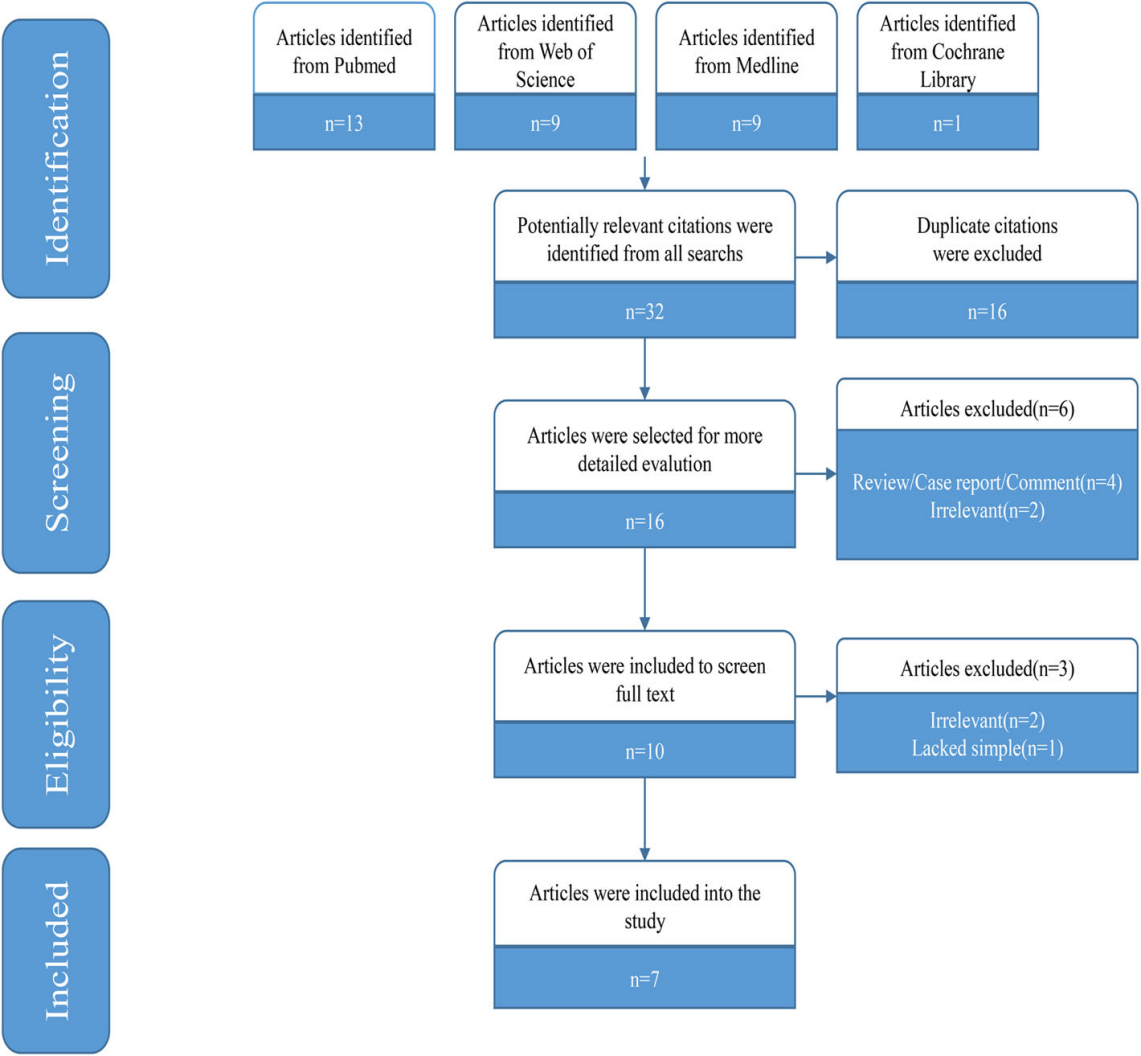
Flow chart of study selection

The initial search yielded 32 records in PubMed, Medline, Web of Science and the Cochrane Library. Of these, 16 duplicate studies were excluded. We excluded 6 records after reading the titles and abstracts. After reviewing the full texts, 7 articles were finally included in this study [11, 12, 23–27]. The selection flowchart of this study is shown in Fig. 1.

In total, 7 studies (ranging from 2008 to 2019) from Asia and Europe (Japan, Germany, and UK) including 8 sets of data, which comprised 405 EC patients were included (sample size median: 45(18–100), mean: 58; CTC-positive patient rate median: 19.7%(13.2–50%)) (Table 1). Based on the sampling time points, 5 studies [11, 12, 25–27] only evaluated CTCs at baseline and two studies [23, 24] evaluated CTCs both at baseline and intra-therapy. HRs for OS was provided in 8 sets of data from 7 studies [11, 12, 23–27], and RFS in 3 sets of data from 3 studies [11, 12, 25], respectively.

**Table 1:** Characteristics of included studies for the meta-analyses

Reference	Year	Country	Patient		Age (years)	Tumor stage	Sampling time	Target antigen/gene	Cutoff	Positive		End point	Treatment regimens
			number	male/female						n	male/female		
Konczalla L *11*	2019	Germany	76	27/19	65(NR)	I—IV	Baseline	CK, CD45	≥1/7.5 ml	15	10/5	OS/RFS	Surgery
Woestemeier A *12*	2018	Germany	45	35/10	63.3	I—III	Baseline	EpCAM, CK (8, 18, 19), CD45	≥1/7.5 ml	7	6/1	OS/RFS	Surgery
Tanaka M *23*	2015	Japan	38	30/8	63 (43–87)	I—IV	Baseline	EpCAM, CK (8, 18, 19), CD45	≥2/7.5 ml	19	14/5	OS	Chemotherapy/Chemoradiotherapy
			38	30/8	63 (43–87)	I—IV	intra-therapy	EpCAM, CK (8, 18, 19), CD45	≥2/7.5 ml	15	NR	OS	
Matsushita D *24*	2015	Japan	90	78/12	65 (46–98)	II—IV	Baseline	EpCAM, CD45	≥1/7.5 ml	25	22/3	OS	Chemotherapy/Chemoradiotherapy
			71	NR	NR	NR	intra-therapy	EpCAM, CD45	≥1/7.5 ml	15	NR	NR	
Reeh M *25*	2015	Germany	100	77/23	66 (32–85)	I—IV	Baseline	EpCAM, CK, CD45	≥1/7.5 ml	18	13/5	OS/RFS	Surgery
Sclafani F *26*	2014	U.K.	18	16/2	61 (38–78)	NR	Baseline	EpCAM, CK CD45	≥2/7.5 ml	8	NR	OS	NR
Hiraiwa K *27*	2008	Japan	38	NR	NR	NR	Baseline	EpCAM, CK CD45	≥2/7.5 ml	5	NR	OS	Chemotherapy

*OS* Overall survival, *DSF* Disease-free survival, *NR* Not reported

### Quality assessment

Among the 7 studies included: 2 studies [26, 27] were of low quality and the other 5 studies [11, 12, 23–25] were of high quality, evaluated based on the NOS (Table 2).

**Table 2:** The assessment of the risk of bias in included studies using the Newcastle–Ottawa scale

Study	Year	Selection (0–4)				Comparability (0–2)		Outcome (0–3)			Total
		REC	SNEC	AE	DO	SC	AF	AO	FU	AFU	
Konczalla L	2019	1	1	1	1	0	0	1	1	0	6
Woestemeier A	2018	1	1	1	1	0	0	1	1	0	6
Tanaka M	2015	1	1	1	1	0	0	1	1	1	7
Matsushita D	2015	1	1	1	1	0	0	1	1	0	6
Reeh M	2015	1	1	1	1	0	0	1	1	0	6
Sclafani F	2014	1	1	0	1	0	0	1	0	0	4
Hiraiwa K	2008	1	1	0	1	0	0	1	0	0	4

*Abbreviations*: *REC* Representativeness of the exposed cohort, *SNEC* Selection of the nonexposed cohort, *AE* Ascertainment of exposure, *DO* Demonstration that outcome of interest was not present at start of study, *SC* Study controls for age, sex, *AF* Study controls for any additional factors (chemoradiotherapy, curative resection), *AO* Assessment of outcome, *FU* Follow-up long enough (36 M) for outcomes to occur, *AFU* Adequacy of follow-up of cohorts (≥90%). “1” means that the study is satisfied the item and “0” means the opposite situation

### Diagnosis

#### CTC detection and clinicopathological features

We extracted and analyzed clinicopathological variables from the included articles in our meta-analysis when they were mentioned in at least 3 studies. The results of the pooled ORs of the parameters of EC patients, which were used to evaluated the potential correlations between the detection of CTCs and clinicopathological parameters, are summarized in Table 3. We extracted and analyzed eight clinicopathological features according to the criteria mentioned above. No significant differences in the results of CTC detection was observed based on age (OR = 1.07 95% CI: 0.62–1.87, I^2^ = 0%) and sex (OR = 1.01, 95% CI: 0.53–1.91, I^2^ = 0%). However, for other clinicopathological parameters, incidence of CTCs was higher among patients with T3/T4 stage (OR = 2.92, 95% CI: 1.31–6.51, I^2^ = 0%) and distant metastasis group (OR = 5.18, 95% CI: 2.38–11.25, I^2^ = 0%) compared to patients with T1/T2 stage or non-metastatic group. Similarly, the pooled results on TNM stage indicated that III/IV group had higher incidence of CTCs compared with I/II group (OR = 1.36, 95% CI: 0.68–2.71, I^2^ = 0%). However, the correlation between the incidence of CTCs and clinical stage was only discussed in three included articles with 221 patients and among them 40 are CTCs-positive. Besides, the studies by Woestemeier A [12] provided the limited data of patients with stage I-III. Therefore, although the results indicated there was no significance of this difference between patients with stage I-II and those with stage III-IV (P = 0.38), with more patients and studies included in the future, the results might suggest significant difference between different clinical status and stages. Interestingly, the AC group had higher incidence of CTCs compared with the SCC group (OR = 1.86, 95% CI: 0.81–4.26, I^2^ = 0%).

**Table 3:** Results of association between CTCs and clinicopathological characteristics

	OR (95% CI)	N	*P*-value
Age: > 65 vs. ≤65(OR)	1.07 (0.62,1.87) I^2^ = 0%	4	0.8
Sex: male vs. female (OR)	1.01 (0.53,1.91) I^2^ = 0%	5	0.98
Histology: AC vs. SCC	1.86 (0.81,4.26) I^2^ = 0%	4	0.14
pT: T3/T4 vs. T1/T2(OR)	2.92 (1.31,6.51) I^2^ = 0%	3	0.009
LN^3^ vs. LN [2](OR)	1.06 (0.61,1.86) I^2^ = 0%	4	0.83
pM:M1 vs. M0(OR)	5.18 (2.38,11.25) I^2^ = 0%	4	< 0.001
Stage: III/IV vs. I/II (OR)	1.36 (0.68,2.71) I^2^ = 0%	3	0.38

*OR* Odds ratio

*P*-value for estimates of OR

“-”: not available

*LN* Lymph node

#### CTC detection and prognosis

**Fig. 2:**
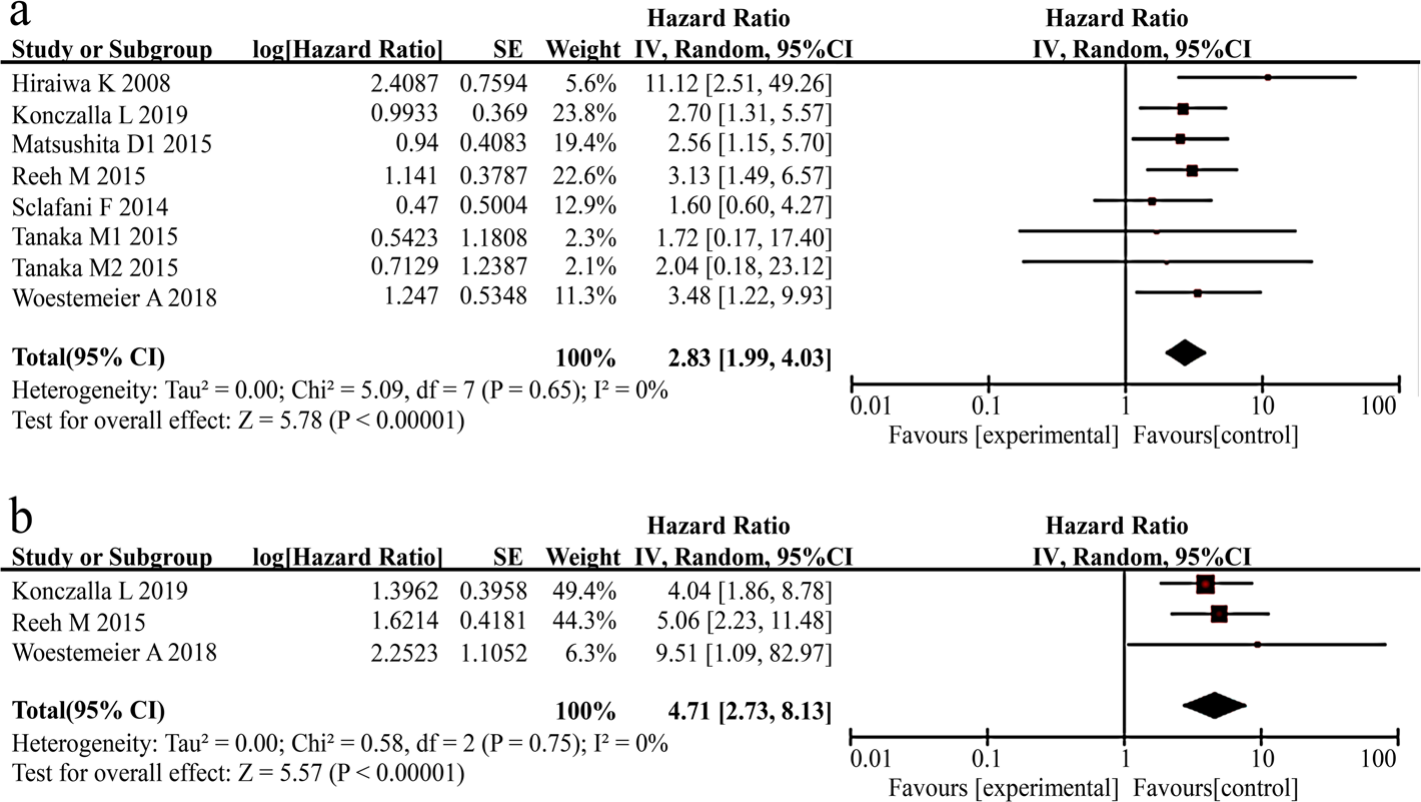
Estimated hazard ratios (HR) summary for OS (**a**) and RFS (b). **a** HR for OS with CTC detection. **b** HR for RFS with CTC detection

To analyse the survival of EC patients, we extracted 8 studies that provided data for OS with 443 samples and 3 studies for RFS with 221 samples. When pooling the HR for OS, an association was observed between CTC-positive status detected in EC patients and poor prognosis for OS, and no significant heterogeneity among these studies was found (HR =2.83, 95% CI: 1.99–4.03, I^2^ = 0%). The results are shown in Fig. 2a. As shown in Fig. 2b, the pooled results showed that an association was observed between CTC detection in EC patients and poor prognosis indicated by RFS (HR = 4.71, 95% CI: 2.73–8.13, I^2^ = 0%).

Furthermore, we performed subgroup analyses according to the differences in the variables (publication year, country, patients’ number, sampling time, cut-off value, CTC positive rate, and quality of the articles). The results are shown in Table 4. The median publication years of the included studies for OS and RFS were 2015 and 2018, respectively. The median number of patients in the OS and RFS studies was 41.5 and 76, respectively. The median positive rates of the patients in the OS and RFS studies were 23.8 and 18%, respectively. The summary analysis results demonstrated significance of CTC positivity as a remarkable prognostic indicator of OS and RFS in most subgroups.

**Table 4:** Results of subgroup analyses on OS and RFS

Variable	OS					RFS				
	n	HR (95% CI)	*P*-value^a^	I^2^	*P*-value^b^	n	HR (95% CI)	*P*-value^a^	I^2^	*P*-value^b^
Year^c^										
> Median	2	2.93 (1.62,5.31)	<0.001	0%	0.7	1	4.04 (1.86,8.78)	<0.001	–	–
≤ Median	6	2.78 (1.79,4.30)	<0.001	0%	43%	2	5.48 (2.54,11.79)	<0.001	0%	0.59
Country										
East Asia	4	3.34 (1.58,7.09)	0.002	12%	0.33	0	–	–	–	–
non-East Asia	4	2.68 (1.76,4.08)	<0.001	0%	0.69	3	4.71 (2.73,8.13)	<0.001	0%	0.75
Patient no.^d^										
>Median	4	2.89 (1.93,4.31)	<0.001	0%	0.96	1	5.06 (2.23,11.48)	<0.001	–	–
≤ Median	4	2.89 (1.03,8.13)	0.04	37%	0.19	2	4.45 (2.15,9.24)	<0.001	0%	0.47
Sampling time										
Baseline	7	2.85 (2.00,4.07)	<0.001	0%	0.54	3	4.71 (2.73,8.13)	<0.001	0%	0.75
intra-therapy	1	2.04 (0.18,23.12)	0.56	–	–	0	–	–	–	–
Cutoff value										
≥ 1/7.5 ml	4	2.89 (1.93,4.31)	<0.001	0%	0.96	3	4.71 (2.73,8.13)	<0.001	0%	0.75
≥ 2/7.5 ml	4	2.89 (1.03,8.13)	0.04	37%	0.19	0	–	–	–	–
Positive rate^e^										
>Median	4	2.09 (1.17,3.74)	0.01	0%	0.91	1	4.04 (1.86,8.78)	<0.001	–	–
≤ Median	4	3.38 (2.17,5.26)	<0.001	0%	0.41	2	5.48 (2.54,11.79)	<0.001	0%	0.59
Quality										
High	7	2.61 (1.82,3.75)	<0.001	0%	0.95	3	4.71 (2.73,8.13)	<0.001	0%	0.75
Low	1	11.12 (2.51,49.26)	–	–	0.17	0	–	–	–	–
Overall	8	2.83 (1.99,4.03)	<0.001	0%	0.65	3	4.71 (2.73,8.13)	<0.001	0%	0.75

^a^*P*-value for estimates of HR.

^b^*P*-value for heterogeneity

^c^ The median year of Os and PFs was 2015 and 2018, respectively

^d^ The median patient number of Os and PFs was 41.5 and 76, respectively

^e^ The median positive rate of Os and PFs was 23.8 and 18%, respectively

“-”: not available

#### CTC detection and DCR

**Fig. 3:**
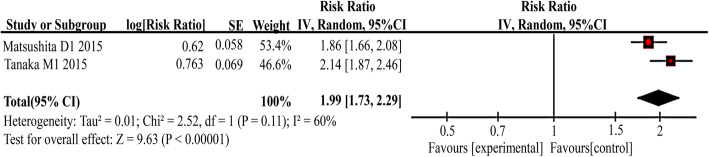
Risk ratio (RR) for DCR

Only 2 studies assessed the association between incidence of CTCs and DCR in patients receiving chemotherapy/chemoradiotherapy, and the overall response rate (ORR) was used to assess the response to chemoradiotherapy. When pooling the estimated RR, CTC-positive patients had a poor response to chemoradiotherapy compared with CTC-negative patients (RR = 1.99, 95% CI: 1.73–2.29, I^2^ = 60%), as shown in Fig. 3.

#### Subgroup analysis and publication bias

**Fig. 4:**
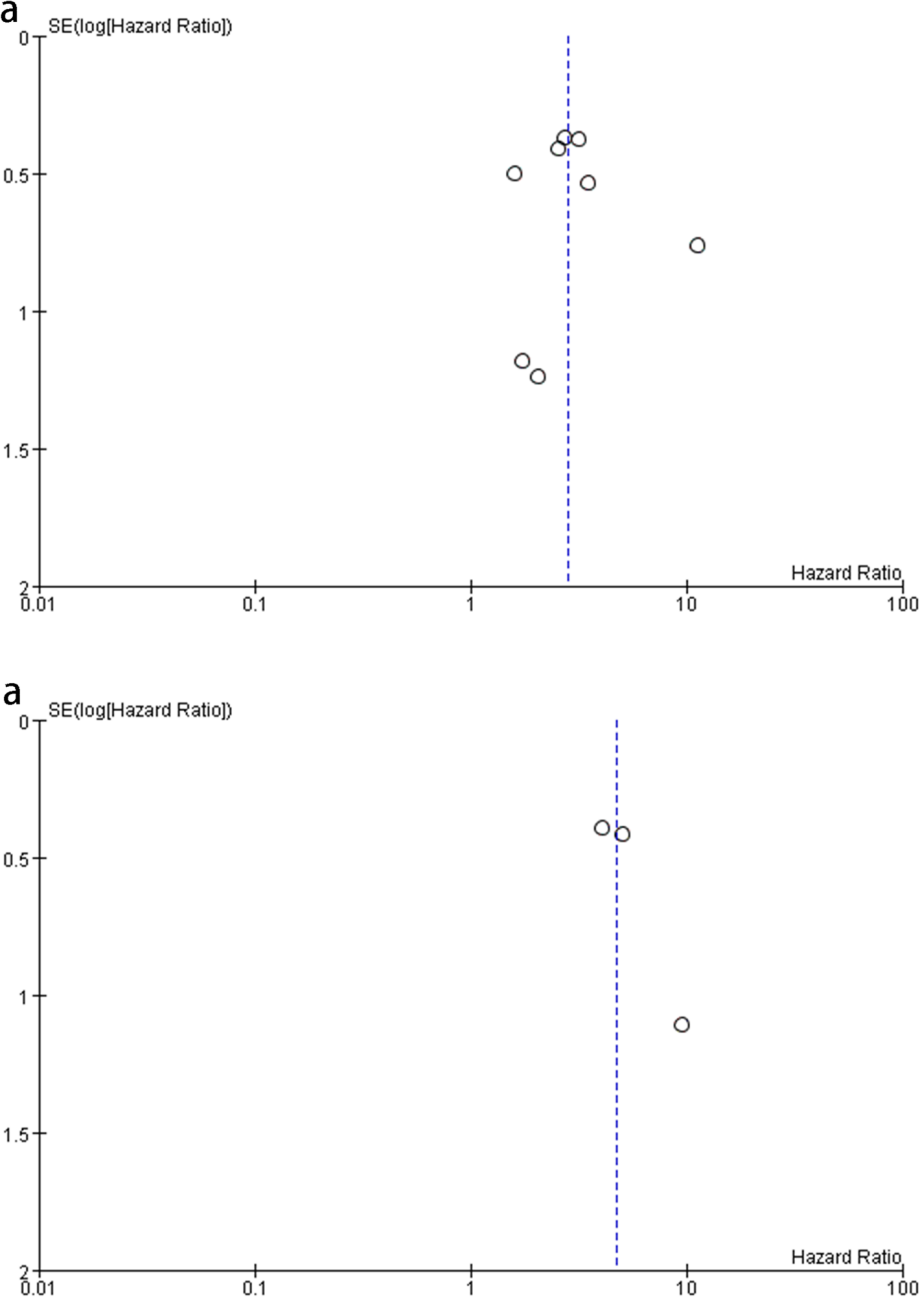
Assessment of publication bias using Funnel plot analysis. **a** Funnel plot analysis of studies on OS. **b** Funnel plot analysis of studies on RFS. Publication bias was not found in the meta-analyses of OS and RFS

Regarding the heterogeneity of the pooled survival effects, there was no statistical significance in between-study heterogeneity for OS and RFS. We used funnel plots to detect publication bias, as shown in Fig. 4. In all comparisons, shape of the funnel plots had a symmetrical distribution. Thus, no significant publication bias was found in the meta-analyses of OS and RFS.

## Discussion

Although the advanced treatment have been widely adopted in EC patients recently, the presence of spreading and recrudescence of EC are still great challenges for both surgeons and patients [3, 28]. Due to late diagnosis and limited treatment options, most EC patients have a poor prognosis and high mortality. To prompt timely diagnosis and treatment, biomarkers to determine the recurrent or metastatic status of EC are in great request. Recently, CTCs, detached cells from a primary tumor in PB, have been increasingly investigated for their prognostic value in many tumors. As described in the “seed and soil” theory [29], CTCs are regarded as critical factors for tumors metastasis [30]. As demonstrated in many studies, high CTCs was associated with the poor survival in many solid tumours, such as breast cancer [31], bladder cancer [32], ovarian cancer and gastric cancer [33, 34]. In addition, due to its benefits of time and cost saving, easy operation and higher specificity and reproducibility, CTC detection from PB can be regarded as an effective evaluation tool for monitoring and assessing treatment effects in EC patients. For EC, several previously published meta-analyses demonstrated the prognostic value of CTCs; however, the assays used to detect CTCs were predominately restricted to polymerase chain reaction (PCR) and immunocytochemistry (ICC) in the included studies [35]. Interestingly, the clinical utility of CTC detection with the CellSearch system from the PB of EC patients has been demonstrated in several studies [11, 12, 23–27]. Thus, to quantitatively assess the clinical value of CTCs determined using the CellSearch system in EC patients is valuable. It is commonly acknowledged that CTCs detected using CellSearch system are EpCAM^high^, and EpCAM^low^ CTCs might be missed due to epithelial-mesenchymal transition (EMT). Researchers have described a method to collect EpCAM^low^ CTCs using immunomagnetic ways to deplete EpCAM^high^ cells, which is favorable for investigating the correlation between EpCAM^low^ CTCs and clinical outcomes of patients [36–38]. Results from a pilot study in patients with metastatic lung cancer did not indicate any significant association between the incidence of EpCAM^low^ CTCs and overall survival (OS). Similar results were found in a research including 97 metastatic non-small-cell lung cancer patients. In other types of cancers such as prostate cancer and breast cancer, the incidence of ≥5 EpCAM^low^ CTCs was not significantly associated with prognosis of patients, contrary to the presence of ≥5 EpCAM^high^. Additionally, previous study also indicated that EpCAM^high^ CTCs from colorectal cancer approximately account for 89% [39], which indicates the numbers of CTCs detected with CellSearch system were more than the missed EpCAM^low^ CTCs, and correspondingly the significance of EpCAM^high^ CTCs were higher. Collectively, these studies all suggested that although CTCs with a mesenchymal phenotype may not be detected by the CellSearch system, obvious significance of EpCAM^low^ CTCs in predicating prognosis in cancer patients was not indicated.

As far as we know, this is the first meta-analysis that focused on the significance of CTC determined only using the CellSearch system in EC. As a result, our results are more informative than those of previous studies. Our meta-analysis of 7 articles including 405 EC patients indicating that CTC-positive patients have poorer OS and RFS compared with CTC-negative patients, which showed that the detection of CTCs has clinicopathological and prognostic value in patients with EC. Moreover, the results of summary analysis demonstrated the significance CTC positivity as a remarkable prognostic indicator of OS and RFS in most subgroups. When pooling the HR for OS, CTC-positive status detected in EC patients was correlated with poor OS (HR = 2.83, 95% CI: 1.99–4.03, I^2^ = 0%) and DFS (HR = 4.71, 95% CI: 2.73–8.13, I^2^ = 0%). Patients with CTC positivity have a worse prognosis than those with CTC negativity. Moon DH suggested that although CTCs detected with the CellSearch system are an independent prognostic marker, it remains to be elucidated whether they can be considered a predictive marker for therapy [40]. However, Riethdorf S indicated that the dynamic monitoring of CTCs with the CellSearch system might help to predict therapeutic efficacy in cancer [41]. Then, we extracted data and analysed the DCR of chemotherapy in patients with EC, and the pooled analysis demonstrated that the DCR of the CTC positivity was lower than that of the CTC negativity (RR = 1.99, 95% CI:1.73–2.29, I^2^ = 60%). Because CTCs could more likely escape from the primary tumour and enter into peripheral blood when the biological control by the primary tumour was not functioning and internal milieu altered, tumour recurrence after surgical treatment was more likely to appear in CTC-positive patients [42]. Therefore, CTC detection can be regarded as an effective evaluation tool for assessing chemoradiotherapy efficacy and monitoring tumor recurrence in many solid tumors [43–45], including EC. Moreover, our meta-analysis demonstrated that CTC positivity was remarkably correlated with TNM staging, pT category, and distant metastasis. EC patients with stage III-IV have higher CTCs-incidence than patients with stage I-II (OR = 1.36). However, the correlation between the incidence of CTCs and clinical stage was only discussed in three included articles with 221 patients and among them 40 are CTCs-positive. Besides, the studies by Woestemeier A [12] provided the limited data of patients with stage I-III. Therefore, although the results indicated there was no significance of this difference between patients with stage I-II and those with stage III-IV (*P* = 0.38), with more patients and studies included in the future, the results might suggest significant difference between different clinical status and stages. Interestingly, the AC group had a notably higher incidence of CTCs compared with the SCC group (OR = 1.86, 95% CI: 0.81–4.26, I^2^ = 0%) the results is not obviously significant (*P* = 0.14), which is consistent with other studies [11]. Besides, studies concerning the relative aggressive behavior of AC group and SCC group is rare. And it is relatively difficult to discuss the correlation between the higher incidence of CTCs and aggressive behavior in AC and SCC group, respectively. In summary, the pooled results indicate that the CTCs determined by the CellSearch system have important clinical value in assessing the prognosis of EC patients, guiding treatment decisions, and monitoring treatment effects. For CTC-positive patients, more early aggressive treatment and effective evaluation may be required.

The CellSearch system used for detection of CTCs has more advantages compared to ICC and PCR, including saving time and cost, easy operation and higher specificity and reproducibility for CTC enrichment. Since our meta-analysis of researches utilizing the CellSearch system for detection of CTCs decreased the heterogeneity caused by various detection assays, there was no statistical significance in between-study heterogeneity for OS and RFS. Therefore, the detection method is the main source of between-study heterogeneity. In all comparisons, shape of the funnel plots had a symmetrical distribution. Thus, no significant publication bias was found in the meta-analyses of OS and RFS.

In addition, clinical consensus still remained equivocal on the optimal cutoff value for predicting the prognosis of EC patients with CTCs. In our meta-analysis, both the cut-off value CTCs ≥1/7.5 ml and CTCs ≥2/7.5 ml seemed to indicate equivalent predicative value, suggesting these two cut-off values are both associated with poor prognosis. However, when we excluded the intra-therapy set of Tanaka et al., a significantly higher HR for OS was found with the cut-off value of CTCs ≥2/7.5 ml (HR = 3.14, 95% CI:1.82–11.97) than with the cut-off value of CTCs ≥1/7.5 ml (HR =2.89, 95% CI: 1.93,4.31). Therefore, in EC patients, the cut-off value of CTCs ≥2/7.5 ml may be correlated with poorer prognosis than the cut-off value of CTCs ≥1/7.5 ml. Thus, high-quality, well-designed, large-scale multi-centre research is needed to identify the better cut-off value and more appropriate sampling time of CTC detection.

Several limitations remained in our study. First, due to several studies didn’t report HRs, the estimated HR was used to assess prognostic effects based on the method described by Tierney et al. [21]. Second, we used extracted data rather than raw data from individual patients, and we could not correct all clinicopathological parameters according to a consistent standard. Third, we limited our analysis to studies published in English, so the choice of language brings another bias. Fourth, the total amount of patients was relatively small in the meta-analysis. Fourth, there are low patients’ number and no multicenter controlled trials in our meta-analysis. Fifth, with the limited data in the included articles, the data considering clinical pathological characteristics and prognosis of AC and SCC group patients were not available separately. Despite these limitations, we still demonstrated that CTC positivity determined using CellSearch system was an indicator of poor prognosis in patients with EC.

## Conclusions

Sum up, our meta-analysis indicated that the presence of CTCs determined using the CellSearch system is correlated with the prognosis of EC patients and provided a scientific foundation for EC staging. Additionally, subgroup analysis indicated that CTC positivity is more associated with a poorer prognosis than CTC negativity. Additionally, the CTCs determined using the CellSearch system can be regarded as an effective evaluation tool for assessing chemoradiotherapy efficacy and monitoring tumour recurrence for EC patients. However, high-quality, well-designed, large-scale multi-centre research is needed to verify our results and confirm the clinical value of CTCs determined using the CellSearch system in EC patients.

## Data Availability

The datasets used and/or analysed during the current study available from the corresponding author on reasonable request.

## Abbreviations

EC: Esophageal carcinoma
CTCs: Circulating tumor cells
PB: Peripheral blood
SCC: Squamous cell carcinoma
AC: Adenocarcinoma
ORs: Odds ratios
RRs: Risk ratios
HRs: Hazard ratios
Cis: Confidence intervals
OS: Overall survival
RFS: Relapse-free survival
NOS: Newcastle-Ottawa Scale
DCR: Disease control rate
CR: Complete response
PR: Partial response
SD: Stable disease
PD: Progressive disease
ORR: Overall response rate
